# The Practitioners' Bookshelf

**Published:** 1893-01-07

**Authors:** 


### THE PRACTITIONERS' BOOKSHELF.

### THE NATIONALISATION OF HEALTH.*

Mr. Havelock Ellis has collected in this volume many striking facts and statistics to show the weak points in our existing medical system. He draws attention in particular to three sources of evil in connection with national health, viz., the want of public control in the prevention of disease, especially with regard to such unhealthy trades as the manufacture of white lead, &c.; secondly, the pauperising influence of hospital treatment; and, thirdly, the deficiencies attaching to the system of private practice. The remarks on private practitioners are manifestly inadequate. If space was lacking for a fuller and fairer discussion of this branch of the subject, it should obviously not have been entered upon at all. But it is certainly true that "not even the most able private practitioner can offer such advantages to his wealthiest patient" as the hospital provides for its poorest. It is a curious anomaly. And it is not surprising that more persons yearly succumb to the temptation of receiving gratis the skill and attention which no money suffices to buy.

Mr. Ellis brings statistics to show that 4,000,000 persons in this country annually are in receipt of free medical relief, and may be properly branded as "paupers." The evil is certainly not less grave from the fact that the ordinary patient has no scruple about accepting the relief, and indeed often no sense of obligation at all, since he is perfectly aware that the great medical schools are dependent on his symptoms.

Mr. Ellis, while guarding himself from the imputation of having "elaborate plans or panaceas to bring forward," looks for a remedy to existing evils in the extension of the hospital system to the whole nation, rich and poor. Since the health of each individual is of incalculable importance to the community, he contends that it should be made a national affair. In the Utopia, then, towards which Mr. Ellis hopes we are tending, the inspection of industries will ensure healthy conditions of work to all; a compulsory registration of all forms of disease will rapidly enlarge the bounds of sanitary science; defective teeth and eyesight will be avoided by the periodic visits of oculist and dentist to all schools, the private practitioner will have disappeared, or practise solely in affiliation with his hospital, while a vast network of hospital organisation, developed on the lines of the poor law infirmary, is to cover the land. Lastly, there will be no more doctors' bills. And as for the hospital rate, Mr. Ellis sanguinely opines that "there are very few households that under a rationally organised system of health, even with the heaviest hospital rate, would not find themselves better off at the end of the year than they are now."

The book is attractively written, and contains much sound criticism and suggestion.

### A MANUAL OF THE OPERATIONS OF SURGERY FOR THE USE OF SENIOR STUDENTS, HOUSE SURGEONS, AND JUNIOR PRACTITIONERS.†

When a text-book has reached a seventh edition the task of a reviewer is comparatively simple, for the facts show that the book is appreciated by those for whom it is intended, and that it supplies a want. This is eminently true of Dr. Joseph Bell's admirable little manual of surgical

* "The Nationalisation of Health." By HAVELOCK ELLIS. (T. Fisher Unwin.)

† "A Manual of the Operations of Surgery for the use of Senior Students, House Surgeons, and Junior Practitioners," Illustrated. By Joseph Bell, M.D., F.R.C.S., Edinburgh. Seventh Edition, Revised and enlarged. (Edinburgh: Oliver and Boyd. 1892).

operations. The choice of operations is good, the account given of them is clear, and not overloaded with details. Within the last two or three years larger and very admirable works on surgical operations have been published, in which the descriptions are much fuller and more detailed than in Dr. Bell's manual. By their very merit these larger works are unfitted for a large class of readers, and to that class Dr. Bell's smaller work will be welcome, and they will find in it a very safe guide.

### HYGIENIC MEASURES IN INFECTIOUS DISEASE.*

This is a small book in which the author sets forth in a plain and condensed form the practical results of recent bacteriology in relation to disinfections and the measures to be taken to prevent the spread of infectious diseases.

It is certainly a difficult matter for the busy practitioner to bear in mind the ever-increasing additions to our knowledge of the infectious diseases. Till recently, the old methods of disinfection were presumed to be efficient, but now that we can isolate, cultivate, and experiment upon the microbes which are known to be the immediate and essential cause of certain diseases, we are able to see clearly how difficult it often is to produce the desired result.

The author has compiled this small book chiefly from the larger works of well-known pathologists who have placed bacteriology on such a firm and scientific footing, and the information conveyed is thoroughly reliable and to the point. It contains many hints of great value to the general practitioner, and its very small dimensions are in many respects a valuable feature. The matter is well arranged, and very convenient for reference.

### INORGANIC CHEMISTRY.†

The reputation of the authors of this favourite manual of chemistry, not only as chemists, but as very successful teachers of chemistry—a matter of greater importance to students—is in itself a sufficient guarantee of excellence. As lecturers on chemistry at a medical school of the highest standing, the authors are well acquainted with the requirements of medical students in the subject, a sound though elementary acquaintance with the chemical properties of the elements and the outlines of chemical theory. A most valuable feature in the work is the introductory section of 83 pages, with chapters on the structure of matter, crystallography, weights and measures, specific gravity, physical behaviour of substances to heat, and to light and electricity; physical properties of solids, liquids, and gases; atoms and molecules, chemical notation, thermo-chemistry, chemical affinity, &c. No knowledge of the subject on the part of the student is assumed, and everything is clearly and concisely put before them, so that with a careful perusal of this section the pupil has been well prepared to clearly comprehend the special properties of chemical substances that the rest of the work deals with. Each element is described on a definite and uniform plan, and this renders it more easy for the student to commit to memory what he reads, and is valuable for reference. A brief description of the physiological action of the elements is included, and this is one of the indications that the special requirements of medical students have been consulted throughout its compilation. We would specially commend this work to students, not so much for the matter it contains—in which it naturally differs but little from other manuals of chemistry which are up to date—as for the remarkably clear and intelligible way in which the information is arranged. This is the distinguishing feature of the author's work, and it will be as greatly appreciated in the future as it has been in the past.

* "Hygienic Measures in Relation to Infectious Diseases." By GEO. H. F. NUTTALL, M.D., Ph.D. Sm. 8vo., pp. 10[illegible]. (G. P. Putnam, New York and London, 1893.)

† A short manual of Inorganic Chemistry. By A. DUPRE Ph.D., F.R.S., F.C.S., &c., and H. WILSON HAKE, Ph.D., F.C.S., F.I.C. Second Edition, Revised. Pp. 365. Illustrated. (Griffin and Co., London. 1892.)

